# Supplementary material for: Prognostic Value of a Novel and Established High-Sensitivity Troponin I Assay in Patients Presenting with Suspected Myocardial Infarction
Source: Biomolecules. 2019 Sep 9;9(9):469. doi: 10.3390/biom9090469 (PMC6769518; doi:10.3390/biom9090469)
Supplement: Supplementary file 1 [file biomolecules-09-00469-s001.pdf]

# Online Supplement: Prognostic Value of a Novel and an Established High-Sensitivity Troponin I Assay in Patients presenting with Suspected Myocardial Infarction

**Nils A. Sörensen [1,2,*,†], Sebastian Ludwig [1,2,*,†], Nataliya Makarova [1,2], Johannes T. Neumann [1,2], Jonas Lehmacher [1], Tau S. Hartikainen [1], Paul M. Haller [1,2], Till Keller [3,4], Stefan Blankenberg [1,2], Dirk Westermann [1,2], Tanja Zeller [1,2,‡] and Niklas Schofer [1,‡]**

[1] Department of General and Interventional Cardiology, University Heart Center, Martinistrasse 52, 20246 Hamburg, Germany
[2] German Center for Cardiovascular Research (DZHK), Partner Site Hamburg/Kiel/Lübeck, Martinistrasse 52, 20246 Hamburg, Germany
[3] Kerckhoff Heart and Thorax Center, Department of Cardiology, Benekestrasse 2–8, 61231 Bad Nauheim, Germany
[4] German Center for Cardiovascular Research (DZHK), Partner Site RheinMain, Benekestrasse 2–8, 61231 Bad Nauheim, Germany
* Correspondence: n.soerensen@uke.de (N.A.S.); se.ludwig@uke.de (S.L.); Tel.: +49-40-7410-59658 (N.A.S.); Fax: +49-40-7410-56599 (N.A.S.)
† Both authors contributed equally as first authors.
‡ Both authors contributed equally as last authors.

**Comparison between high-sensitivity troponin I assays**

Both assays (Method A: Abbott, Method B: Singulex) were compared using Passing-Bablok (**Figure S3A–C**) and Bland-Altman (**Figure S4A–C**) regression. Regression analysis was performed for the overall patient cohort (**Figures S3 and S4A**) as well as for the subgroups of AMI patients (**Figures S3 and S4B**) and non-AMI patients (**Figures S3 and S4C**). Bias between both methods was 0.7844 (standard deviation (SE): 0.5710) for the overall patient cohort, 0.7325 (SE: 0.5710) for the subgroup of AMI patients and 0.7993 (SE: 0.5710) for the subgroup of non-AMI patients.

**Table 1.** Patient characteristics according to thirds of troponin levels analyzed by both assays.

| | 1st Third Singulex (*N* = 780) | 1st Third Abbott (*N* = 780) | 2nd Third Singulex (*N* = 775) | 2nd Third Abbott (*N* = 776) | 3rd Third Singulex (*N* = 777) | 3rd Third Abbott (*N* = 776) |
|---|---|---|---|---|---|---|
| Age (years) | 53.0 (43.0, 63.6) | 54.0 (44.4, 66.0) | 69.0 (59.0, 76.0) | 68.0 (55.0, 76.0) | 69.0 (57.0, 76.0) | 69.0 (57.0, 77.0) |
| Sex (male) No. (%) | 435 (55.8) | 436 (55.9) | 518 (66.8) | 520 (67.0) | 555 (71.4) | 552 (71.1) |
| Current smoker No. (%) | 228 (29.4) | 214 (27.5) | 136 (17.6) | 159 (20.6) | 203 (26.4) | 194 (25.2) |
| Diabetes No. (%) | 62 (8.1) | 61 (7.9) | 126 (16.4) | 127 (16.6) | 146 (19.0) | 146 (19.0) |
| BMI (kg/m$^2$) | 25.8 (23.3, 28.9) | 25.9 (23.2, 29.0) | 27.0 (24.4, 30.7) | 26.9 (24.5, 30.2) | 26.8 (24.2, 30.1) | 26.8 (24.2, 30.4) |
| HTN No. (%) | 372 (47.8) | 406 (52.1) | 627 (81.2) | 595 (77.0) | 611 (78.7) | 609 (78.6) |
| HLP No. (%) | 273 (35.0) | 289 (37.1) | 401 (51.7) | 394 (50.8) | 455 (58.6) | 446 (57.5) |
| History of AMI No. (%) | 46 (5.9) | 53 (6.8) | 164 (21.2) | 156 (20.2) | 202 (26.1) | 203 (26.3) |
| History of CAD No. (%) | 121 (15.6) | 136 (17.6) | 324 (42.0) | 314 (40.7) | 346 (44.5) | 341 (43.9) |
| Family history of CAD No. (%) | 205 (26.9) | 205 (26.9) | 159 (21.3) | 162 (21.5) | 157 (20.9) | 154 (20.6) |
| AF No. (%) | 58 (7.5) | 69 (8.9) | 158 (20.5) | 144 (18.7) | 166 (21.5) | 169 (21.9) |
| HF No. (%) | 17 (2.2) | 14 (1.8) | 72 (9.3) | 80 (10.4) | 128 (16.6) | 123 (16.0) |
| eGFR (mL/min for 1.73m$^2$) | 87.9 (75.6, 100.1) | 86.9 (74.1, 99.0) | 73.4 (59.5, 86.7) | 74.9 (60.5, 87.7) | 69.4 (52.0, 85.8) | 69.3 (52.3, 86.0) |
| GRACE > 140 No. (%) | 6 (0.8) | 4 (0.5) | 80 (10.6) | 82 (10.8) | 159 (21.4) | 159 (21.5) |
| Troponin I (ng/L) | 0.7 (0.5, 1.0)* | 2.3 (1.8, 3.2)$^+$ | 2.3 (1.8, 3.4)* | 6.8 (5.3, 9.7)$^+$ | 32.8 (10.4, 282.4)* | 81.8 (25.4, 552.9)$^+$ |
| Final Diagnosis of AMI | 6 (0.8) | 11 (1.4) | 59 (7.6) | 61 (7.9) | 453 (58.3) | 446 (57.5) |

Baseline characteristics of patients divided by thirds of troponin levels according to both assays are presented as absolute and relative frequencies for categorical variables and quartiles for continuous variables. * indicates levels analyzed by Singulex, $^+$ indicates levels analyzed by Abbott. AMI stands for acute myocardial infarction. eGFR stands for estimated Glomerular Filtration Rate. GRACE stands for Global Registry of Acute Coronary Events.

**Table 2.** Patient characteristics of patients with the final diagnosis of AMI according to thirds of troponin levels analyzed by both assays.

| | **1st Third Singulex (*N* = 780)** | **1st Third Abbott (*N* = 780)** | **2nd Third Singulex (*N* = 775)** | **2nd Third Abbott (*N* = 776)** | **3rd Third Singulex (*N* = 777)** | **3rd Third Abbott (*N* = 776)** |
|---|---|---|---|---|---|---|
| Age (years) | 68.0 (58.0, 75.3) | 68.0 (58.0, 75.3) | 67.5 (58.0, 75.6) | 68.0 (58.0, 75.0) | 68.0 (56.7, 75.0) | 68.0 (57.0, 75.3) |
| Sex (male) No. (%) | 118 (68.2) | 119 (68.8) | 124 (72.1) | 123 (71.5) | 126 (72.8) | 126 (72.8) |
| Current smoker No. (%) | 49 (28.3) | 50 (28.9) | 48 (28.2) | 50 (29.2) | 64 (37.0) | 61 (35.5) |
| Diabetes No. (%) | 32 (18.7) | 33 (19.3) | 40 (23.5) | 41 (24.1) | 23 (13.4) | 21 (12.2) |
| BMI (kg/m$^2$) | 26.8 (23.6, 29.8) | 26.9 (23.5, 29.9) | 27.5 (24.5, 30.1) | 27.3 (24.7, 30.2) | 26.2 (24.0, 29.0) | 26.1 (23.9, 28.9) |
| HTN No. (%) | 137 (79.7) | 135 (78.5) | 135 (78.5) | 136 (79.1) | 125 (72.3) | 126 (72.8) |
| HLP No. (%) | 82 (47.4) | 82 (47.4) | 103 (59.9) | 107 (62.2) | 102 (59.0) | 98 (56.6) |
| History of AMI No. (%) | 37 (21.4) | 35 (20.2) | 41 (23.8) | 44 (25.7) | 34 (19.9) | 33 (19.2) |
| History of CAD No. (%) | 68 (39.3) | 66 (38.2) | 71 (41.3) | 75 (43.6) | 61 (35.3) | 59 (34.1) |
| Family history of CAD No. (%) | 31 (18.5) | 32 (19.0) | 40 (24.0) | 39 (23.5) | 39 (23.8) | 39 (23.6) |
| AF No. (%) | 37 (21.5) | 35 (20.3) | 26 (15.3) | 28 (16.5) | 18 (10.4) | 18 (10.4) |
| HF No. (%) | 24 (13.9) | 21 (12.1) | 19 (11.1) | 19 (11.2) | 21 (12.3) | 24 (14.0) |
| eGFR (mL/min for 1.73m$^2$) | 69.2 (55.3, 83.3) | 70.0 (56.9, 83.6) | 69.5 (53.9, 84.4) | 67.5 (51.5, 83.7) | 72.0 (56.0, 89.4) | 72.8 (55.0, 89.3) |
| GRACE > 140 No. (%) | 39 (23.4) | 36 (21.7) | 31 (18.6) | 31 (18.5) | 26 (16.0) | 29 (17.8) |
| Troponin I (ng/L) | 8.0 (3.6, 13.9) * | 16.9 (8.8, 26.8) $^+$ | 88.3 (48.2, 175.2) * | 182.8 (103.2, 329.0) $^+$ | 1401.5 (612.6, 4041.3) * | 3207.4 (1381.9, 10392.6) $^+$ |

Baseline characteristics of patients with a final diagnosis of AMI divided by thirds of troponin levels according to both assays are presented as absolute and relative frequencies for categorical variables and quartiles for continuous variables. * indicates levels analyzed by Singulex, $^+$ indicates levels analyzed by Abbott. AMI stands for acute myocardial infarction. eGFR stands for estimated Glomerular Filtration Rate. GRACE stands for Global Registry of Acute Coronary Events.

**Table 3.** Patient characteristics of patients with the final diagnosis of non-AMI according to thirds of troponin levels analyzed by both assays.

| | 1st Third Singulex (*N* = 780) | 1st Third Abbott (*N* = 780) | 2nd Third Singulex (*N* = 775) | 2nd Third Abbott (*N* = 776) | 3rd Third Singulex (*N* = 777) | 3rd Third Abbott (*N* = 776) |
|---|---|---|---|---|---|---|
| Age (years) | 50.0 (42.0, 60.0) | 53.0 (43.0, 63.8) | 66.0 (55.0, 74.0) | 66.0 (52.0, 74.0) | 70.0 (59.0, 77.0) | 70.0 (58.0, 77.6) |
| Sex (male) No. (%) | 323 (53.2) | 324 (53.0) | 394 (65.3) | 394 (65.8) | 423 (70.0) | 422 (69.9) |
| Current smoker No. (%) | 189 (31.2) | 175 (28.7) | 113 (18.9) | 115 (19.4) | 104 (17.4) | 116 (19.4) |
| Diabetes No. (%) | 40 (6.7) | 45 (7.5) | 79 (13.2) | 82 (13.8) | 120 (20.2) | 112 (18.8) |
| BMI ($kg/m^2$) | 25.4 (22.9, 28.7) | 25.7 (23.1, 28.8) | 27.1 (24.6, 30.4) | 26.8 (24.4, 30.1) | 26.8 (24.5, 30.7) | 26.8 (24.5, 30.7) |
| HTN No. (%) | 262 (43.2) | 302 (49.5) | 449 (74.8) | 418 (70.1) | 502 (83.1) | 493 (81.6) |
| HLP No. (%) | 194 (32.0) | 212 (34.7) | 294 (48.8) | 291 (48.6) | 354 (58.6) | 339 (56.1) |
| History of AMI No. (%) | 25 (4.1) | 31 (5.1) | 94 (15.7) | 97 (16.3) | 181 (30.1) | 172 (28.5) |
| History of CAD No. (%) | 73 (12.1) | 96 (15.8) | 200 (33.4) | 198 (33.3) | 318 (52.7) | 297 (49.3) |
| Family history of CAD No. (%) | 167 (28.0) | 162 (27.2) | 134 (23.0) | 137 (23.5) | 110 (18.9) | 112 (19.2) |
| AF No. (%) | 34 (5.6) | 51 (8.4) | 92 (15.3) | 87 (14.7) | 175 (29.2) | 163 (27.1) |
| HF No. (%) | 11 (1.8) | 12 (2.0) | 33 (5.5) | 31 (5.2) | 109 (18.2) | 110 (18.3) |
| eGFR (mL/min for $1.73m^2$) | 89.8 (77.8, 102.0) | 88.0 (75.7, 100.3) | 78.2 (64.1, 89.2) | 78.5 (64.9, 90.8) | 68.3 (50.8, 84.4) | 70.1 (51.8, 86.2) |
| GRACE > 140 No. (%) | 1 (0.2) | 3 (0.5) | 29 (4.9) | 30 (5.2) | 119 (20.6) | 116 (20.0) |
| Troponin I (ng/L) | 0.6 (0.5, 0.8)* | 1.9 (1.8, 2.6)$^+$ | 1.6 (1.3, 2.1)* | 5.0 (4.1, 6.1)$^+$ | 5.8 (3.8, 11.8)* | 15.2 (10.6, 32.8)+ |

Baseline characteristics of patients with a final diagnosis of non-AMI divided by thirds of troponin levels according to both assays are presented as absolute and relative frequencies for categorical variables and quartiles for continuous variables. * indicates levels analyzed by Singulex, $^+$ indicates levels analyzed by Abbott. AMI stands for acute myocardial infarction. eGFR stands for estimated Glomerular Filtration Rate. GRACE stands for Global Registry of Acute Coronary Events.

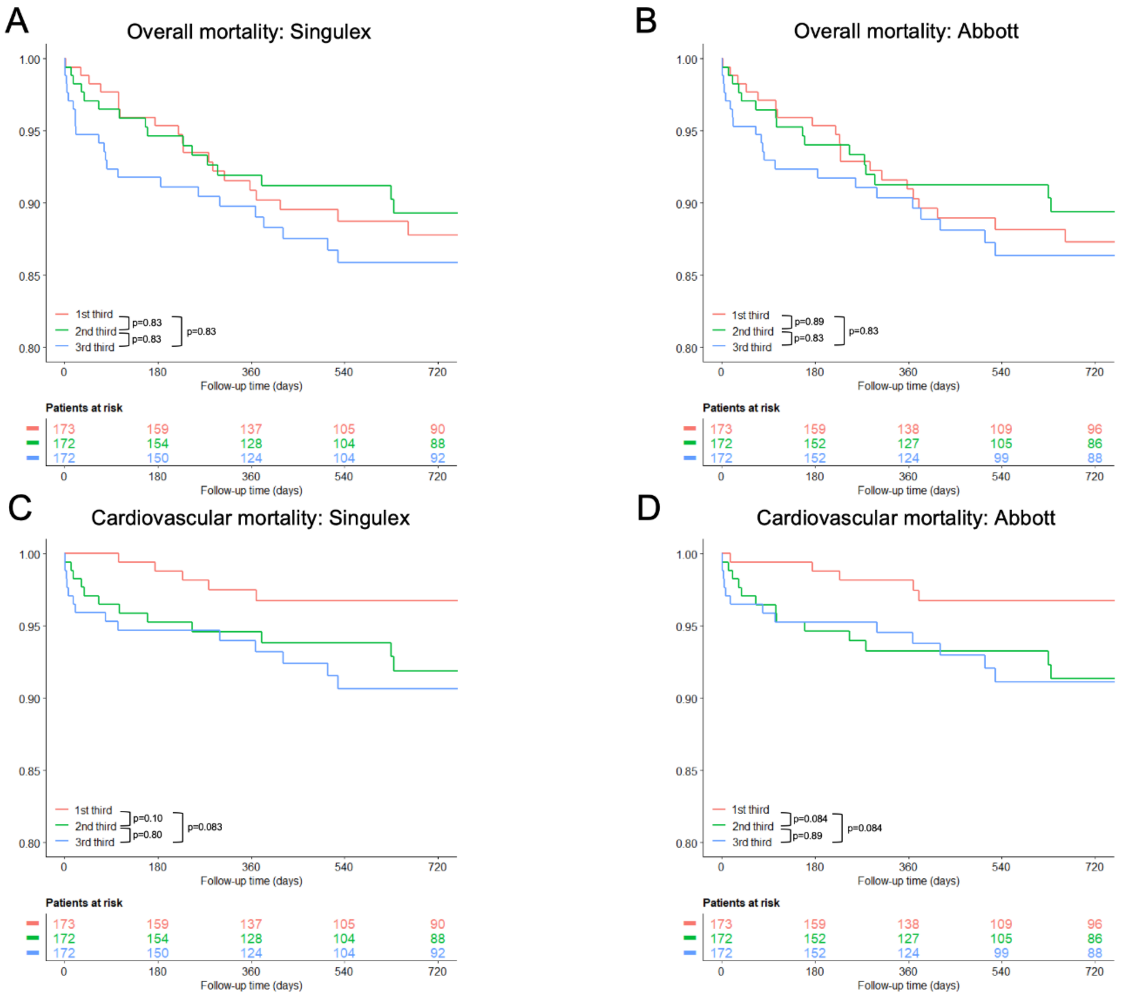


**Figure 1.** Survival analysis according to thirds of troponin levels in patients with AMI. Kaplan–Meier curves according thirds for the endpoints overall (A+B) and cardiovascular mortality (C+D) in patients with adjudicated diagnosis of AMI. P stands for p-value of the log-rank test.

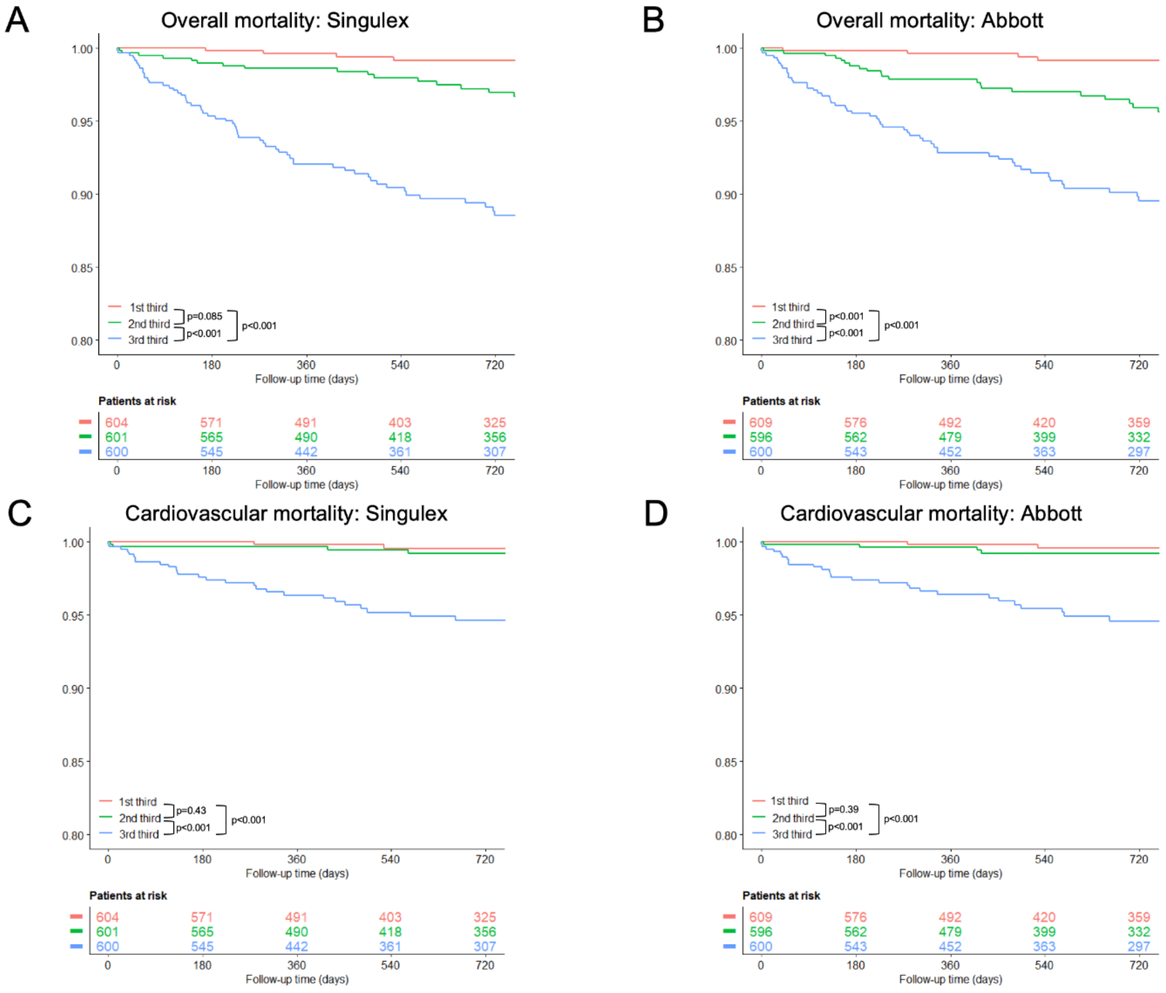


**Figure 2.** Survival analysis according to thirds of troponin levels in patients without AMI. Kaplan–Meier curves according thirds for the endpoints overall (A+B) and cardiovascular mortality (C+D) in patients with adjudicated diagnosis of non-AMI. P stands for p-value of the log-rank test.

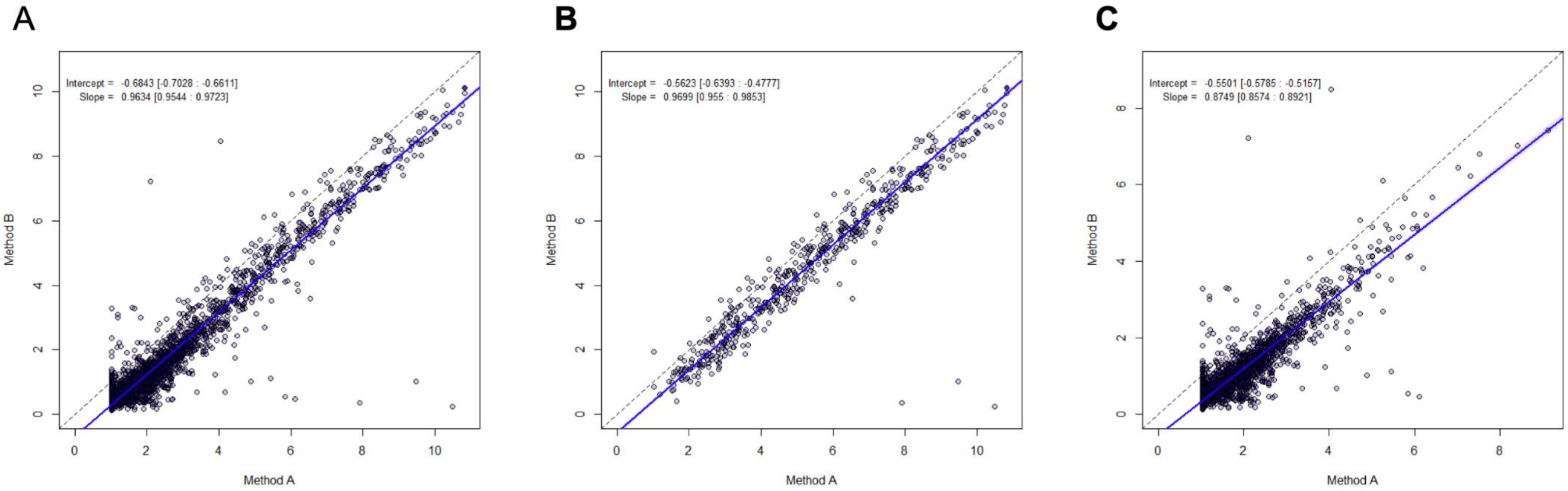


**Figure 3.** Correlation of both hs-TnI assays.

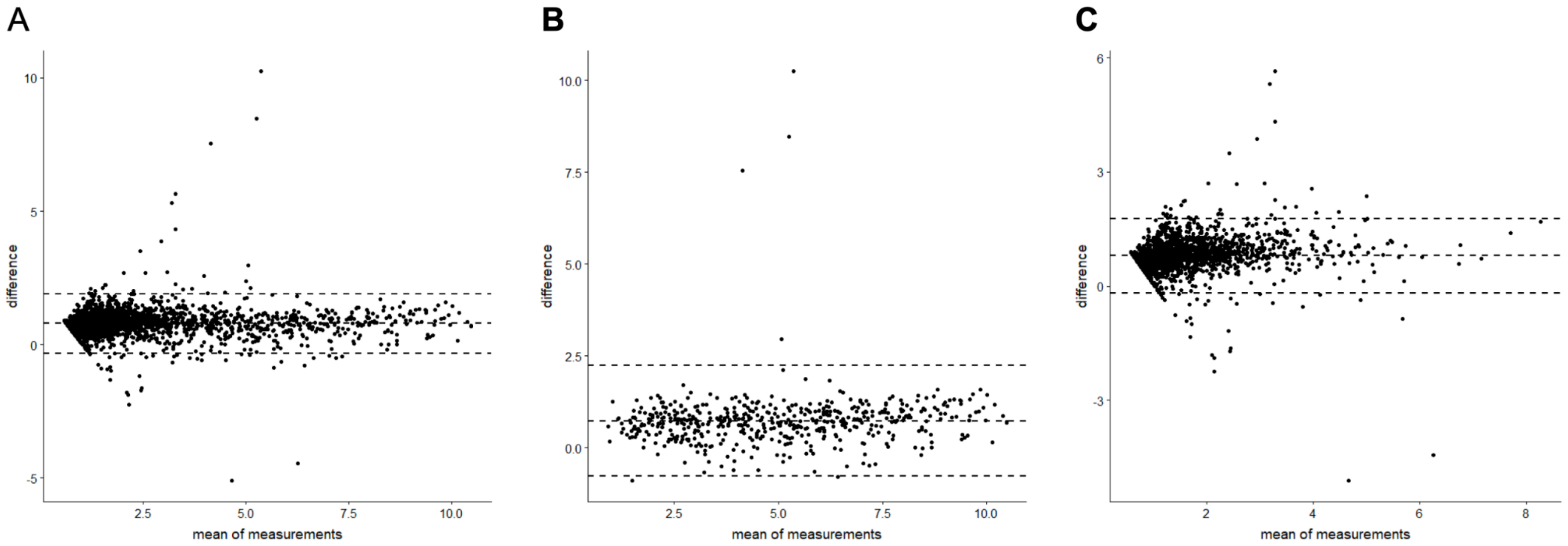


**Figure 4.** Bland-Altman analysis for comparison of both hs-TnI assays.
